# Supplementary material for: Real-time genomic pathogen, resistance, and host range characterization from passive water sampling of wetland ecosystems
Source: Appl Environ Microbiol. 2026 Apr 24;92(5):e02543-25. doi: 10.1128/aem.02543-25 (PMC13188864; doi:10.1128/aem.02543-25)
Supplement: Supplemental material — Descriptive legends for Tables S1 to S4; Fig. S1 to S4. [file aem.02543-25-s0001.docx]

## Supplementary Information

**Supplementary Table 1.** **DNA metagenomics metrics of all samples**, including total DNA yield and concentration, total number of sequencing reads, number of sequencing reads after filtering, read length distribution (median and N50), read-based taxonomic classifications, *de novo* assembly statistics using metaFlye and nanoMDBG (number of contigs, number of bases in contigs, median contig length, and N50 contig), Prokka annotations, and contig-based taxonomic classifications (Methodology).

**Supplementary Table 2.** **RNA virome metrics of all samples**, including RNA concentration and total yield, and cDNA concentration and total yield post-SMART-9N, and total number of sequencing reads, read length distribution (median and N50), viral mapping results using DIAMOND blastx (number of reads and contigs mapping to viral kingdom and families, and percentage of reads mapping to viruses), and assembly statistics using nanoMDBG (number of contigs, number bases in contigs, median contig length, and N50 contig) (Methodology).

**Supplementary Table 3.** **Pathogen species detection of all samples based on sequencing reads**: sampling site, sample ID, pathogen species, and number of reads mapping to each pathogen (Methodology).

**Supplementary Table 4. Pathogen species detection of all samples based on nanoMDBG assemblies**: sampling site, sample ID, pathogen species, and number of contigs mapping to each pathogen (Methodology).

**Supplementary Figure 1. Sampling locations and tools. (A)** Geographic overview of the twelve sampled wetland sites along the East Atlantic Flyway. Anthropogenically impacted sites are indicated by red stars and natural wetlands by red circles. The main migratory corridor of the East Atlantic Flyway is shown as a yellow line. The interactive Google Earth map can be found here: <https://www.bit.ly/locations_pathogen>. **(B)** Photos of the 3D-printed torpedo-shaped passive water samplers.

**Supplementary Figure 2. Quality control summary of DNA and RNA sequencing data.** The boxplots compare **(A)** nucleic acid yield, **(B)** total raw reads, and **(C)** median read length between DNA- and RNA-based sequencing libraries. Histograms show the sequencing read length distribution for all **(D)** DNA, and **(E)** cDNA reads, with dashed vertical lines indicating the median read lengths. **(F)** Number of total metagenomic reads after filtering and subset of reads mapping to the phylum and genus levels and to the top 20 genera. The horizontal dashed line indicates the downsampling threshold of 87,000 reads. GA3.1 was excluded for subequent analyses since it did not reach the downsampling threshold.

**Supplementary Figure 3. Comparison of *de novo* metagenome assemblers.** Comparison of metaFlye and nanoMDBG long-read assemblers across all samples in terms of **(A)** number of assembled contigs, **(B)** number of assembled bases, **(C)** median contig length, **(D)** contig N50 value, **(E)** number of unique detected species, and **(F)** number of gene annotations.

**Supplementary Figure 4. AIV phylogenetic tree.** Phylogenetic analysis of the AIV HA H4 segments from our sample FA2 (highlighted in red) with H4 segments from the GISAID dataset in Europe from January 2015 to August 2025. A branch length of 0.01 corresponds to ~1 substitution per 100 sites. The colour scale represents the country where the viruses were collected.
